# Monitoring of muscle mass in critically ill patients: comparison of ultrasound and two bioelectrical impedance analysis devices

**DOI:** 10.1186/s40560-019-0416-y

**Published:** 2019-12-16

**Authors:** Nobuto Nakanishi, Rie Tsutsumi, Yoshihiro Okayama, Takuya Takashima, Yoshitoyo Ueno, Taiga Itagaki, Yasuo Tsutsumi, Hiroshi Sakaue, Jun Oto

**Affiliations:** 1 Emergency and Critical Care Medicine, Tokushima University Hospital, 2-50-1 Kuramoto, Tokushima, 770-8503 Japan; 2 Department of Nutrition and Metabolism, Tokushima University Graduate School of Biomedical Sciences, 3-18-15 Kuramoto, Tokushima, 770-8503 Japan; 3 Clinical Trial Center for Developmental therapeutics, Tokushima University Hospital, 2-50-1 Kuramoto, Tokushima, 770-8503 Japan; 4 Department of Anesthesiology and Critical Care, Hiroshima University, 1-2-3 Kasumi, Hiroshima, 734-8551 Japan; 5 Emergency and Disaster Medicine, Tokushima University Hospital, 2-50-1 Kuramoto, Tokushima, 770-8503 Japan

**Keywords:** Muscle mass, Ultrasound, Bioelectrical impedance analysis, Fluid balance, Critically ill patients

## Abstract

**Background:**

Skeletal muscle atrophy commonly occurs in critically ill patients, and decreased muscle mass is associated with worse clinical outcomes. Muscle mass can be assessed using various tools, including ultrasound and bioelectrical impedance analysis (BIA). However, the effectiveness of muscle mass monitoring is unclear in critically ill patients. This study was conducted to compare ultrasound and BIA for the monitoring of muscle mass in critically ill patients.

**Methods:**

We recruited adult patients who were expected to undergo mechanical ventilation for > 48 h and to remain in the intensive care unit (ICU) for > 5 days. On days 1, 3, 5, 7, and 10, muscle mass was evaluated using an ultrasound and two BIA devices (Bioscan: Malton International, England; Physion: Nippon Shooter, Japan). The influence of fluid balance was also evaluated between each measurement day.

**Results:**

We analyzed 93 images in 21 patients. The age of the patients was 69 (interquartile range, IQR, 59–74) years, with 16 men and 5 women. The length of ICU stay was 11 days (IQR, 9–25 days). The muscle mass, monitored by ultrasound, decreased progressively by 9.2% (95% confidence interval (CI), 5.9–12.5%), 12.7% (95% CI, 9.3–16.1%), 18.2% (95% CI, 14.7–21.6%), and 21.8% (95% CI, 17.9–25.7%) on days 3, 5, 7, and 10 (*p* <  0.01), respectively, with no influence of fluid balance (*r* = 0.04, *p* = 0.74). The muscle mass did not decrease significantly in both the BIA devices (Bioscan, *p* = 0.14; Physion, *p* = 0.60), and an influence of fluid balance was observed (Bioscan, *r* = 0.37, *p* <  0.01; Physion, *r* = 0.51, *p* <  0.01). The muscle mass assessment at one point between ultrasound and BIA was moderately correlated (Bioscan, *r* = 0.51, *p* <  0.01; Physion, *r* = 0.37, *p* <  0.01), but the change of muscle mass in the same patient did not correlate between these two devices (Bioscan, *r* = − 0.05, *p* = 0.69; Physion, *r* = 0.23, *p* = 0.07).

**Conclusions:**

Ultrasound is suitable for sequential monitoring of muscle atrophy in critically ill patients. Monitoring by BIA should be carefully interpreted owing to the influence of fluid change.

**Trial registration:**

UMIN000031316. Retrospectively registered on 15 February 2018.

## Introduction

Skeletal muscle atrophy commonly occurs in critically ill patients. The muscles of critically ill patients can rapidly atrophy after admission in the intensive care unit (ICU). During intensive care, a noticeable reduction in muscle mass, starts within 3 days and progressively worsens thereafter [1]. Our previous report suggested that the upper- and lower-limb muscle mass of intensive care patients decrease by 13–21% within 7 days of admission [2]. Moreover, decreased muscle mass is associated with prolonged weaning from mechanical ventilation and length of ICU stay, as well as higher mortality [3].

Although monitoring of muscle mass is important, identification of an optimal method of estimating muscle mass remains difficult. There are many methods that can estimate muscle mass. In recent years, the use of ultrasound and bioelectrical impedance analysis (BIA) has become more widely accepted in the ICU [4]. A recent study by Kim et al. reported that BIA could be used for whole-body muscle mass assessment at one point in critically ill patients [5]. However, sequential muscle mass monitoring capability of BIA in the same critically ill patients, not muscle mass assessment at one point, has not been investigated. Patients in the ICU are generally under abnormal fluid status. Because BIA indirectly estimates muscle mass using electrical resistance, and the resistance is affected by fluid status, the hypothesis that BIA could not accurately monitor the change of muscle mass in critically ill patients is reasonable.

This study aimed to compare the use of ultrasound and BIA for muscle mass monitoring in critically ill patients. The influence of fluid shift was also analyzed because edema may complicate these measurements and few studies cleared the complication. In addition, muscle mass was retrospectively evaluated using computed tomography (CT) to compare with previous studies.

## Materials and methods

### Study design and setting

We conducted a prospective observational single-center study at the mixed medical/surgical ICU of Tokushima University Hospital from May 2016 to May 2017. This study was approved by the clinical research ethics committee at Tokushima University Hospital (approval number 2593). At enrollment, written informed consent was obtained from patients or from an authorized surrogate.

### Study population

We consecutively recruited adult patients who were expected to require mechanical ventilation for > 48 h and to remain in the ICU for > 5 days. The patients were prospectively recruited within 24 h of ICU admission. We excluded patients who fell under one or more of the following categories: age younger than 18 years; pregnant; exhibiting trauma to or amputation of upper or lower limbs; diagnosed with primary neuromuscular disease; embedded electronic devices, such as a pacemaker or implanted cardiac defibrillator because the current from BIA could affect the device activity.

### Ultrasonographic measurement

For sonography, we used a HI VISION Preirus with B-mode imaging, which was connected to a 6.5 MHz 3.8-cm linear transducer (EUP-L73S Features; both Hitachi Medical Corporation, Tokyo, Japan). All scanning was performed with patients in the supine position and elbows and knees in passive extension. Generous amounts of contact gel were applied to avoid compression of the muscles by the transducer. The transducer was placed perpendicular relative to the long axis of the limbs. The sum of biceps brachii and rectus femoris cross-sectional area was used for the comparison because of the improved whole-body muscle mass assessment with the sum of the upper and lower limb [6]. The cross-sectional area at the dominant limb was evaluated on days 1, 3, 5, 7, and 10 (Additional file 1: Figs. S1, S2). The subcutaneous tissue thickness was also evaluated for the assessment of edema (Additional file 1: Fig. S2). The sum of subcutaneous tissue thickness at the biceps brachii and rectus femoris muscles was used for the assessment. Before commencing the study, the intra- and inter-observer reproducibilities were 0.93–0.99 and 0.93–0.98, and 0.83–0.99 and 0.90–0.99 for the muscle and subcutaneous tissue, respectively, as assessed by two ICU physicians (Additional file 1: Table S1). Therefore, each measurement was performed by the same investigator. The investigator performing measurements was not blinded to patients’ condition, but image analyses were blinded by concealing patients’ name and measurement days.

### Bioelectrical impedance analysis

BIA is a noninvasive tool that measures impedance by sending a weak electric current through the body. BIA indirectly estimates body composition, including fat, water, bone mass, lean body mass, and muscle. BIA was used on days 1, 3, 5, 7, and 10. Two different BIA devices were used as follows: Bioscan 920-2 (Malton International, Essex, United Kingdom) and Physion MD (Nippon Shooter Ltd., Tokyo, Japan). BIA measurements were also conducted on days 1, 3, 5, 7, and 10, with the patients in the supine position with extended limbs and 30° abduction of the shoulder and hip.

#### Bioscan 920-2

Bioscan can measure muscle mass by using four to eight electrodes. We used four electrodes for hemi-lateral upper and lower extremities. The electrodes were attached to the dorsum of the wrists and third metacarpi of the hand, and the anterior surface of the ankles and third metacarpi of the foot. Bioscan has multi-frequency measurement (5, 50, 100, and 200 kHz) with low-amplitude current (700 μA). The range of impedance is 2–1200 Ω. The data obtained included total body water, intracellular water, extracellular water, and skeletal muscle mass.

#### Physion MD

Physion MD can measure site-specific muscle mass, including the upper arms, forearms, thighs, lower legs, and trunk by using a four-limb 12-lead electrode. The measuring frequency and current are 50 kHz and 500 μA, respectively. The range of impedance is 10–1500 Ω. The data obtained included total body water (no data regarding intra- or extracellular water) and skeletal muscle mass.

### Computed tomography

We retrospectively evaluated the muscle mass using the transverse plane in CT because previous studies have compared the muscle mass assessment between BIA and CT [5, 7]. CT examinations were performed for clinical purpose. Therefore, the examination day is not consistent among included patients. The muscle mass at the L3 spine level correlates well with whole-body muscle mass. Therefore, the muscle mass area at L3 was measured by outlining the muscle area using Image J software (National Institutes of Health, Bethesda, MD, USA, Additional file 1: Fig. S3).

### Fluid balance

Fluid balance was evaluated during ICU stay because the fluid status may interfere with the assessment of muscle mass. Fluid intake included intravenous fluids, total parenteral and enteral nutrition, blood products, and intravenous medications. Fluid output included urine, excrement, blood loss, output from drains and other body cavities, and gastric aspirate. Other fluid losses, such as sweat and respiratory evaporation, were excluded. We used fluid balance from a measurement day to the day before the next measurement. We compared the fluid balance with the change of measured variables in the same intervals. Additionally, the change of balance was analyzed from the admission to each measurement days. Fluid balance was classified into interval fluid balance between each measurement day or accumulated fluid balance from day 1 to measurement days (Additional file 1: Fig. S4).

### Outcomes

The primary outcome was the time course of muscle atrophy, evaluated using ultrasound and two BIA devices. To assess the changes in muscle mass, we calculated the atrophy rate, defined as the percent variation of muscle mass as compared with values on day 1.

The secondary outcome was a comparison of muscle mass assessment between ultrasound and BIA. We analyzed the muscle mass assessment at one point and subsequent muscle mass change from day 1 to each study day. Then, we retrospectively compared these muscle mass assessment methods with CT evaluation in a limited number of patients. Finally, we evaluated the association of these measurement methods and fluid balance.

### Statistical analyses

Mixed-effects models for repeated measures (MMRM) was used to assess the changes in muscle mass over time: statistical significance of muscle mass atrophy at each time point was also tested using 95% confidence intervals (CIs), with intervals excluding zero considered to be statistically significant. Data were presented as mean (95% CI). The Pearson correlation coefficient was used to investigate relationships among measured variables. Due to the exploratory research, the sample size was not calculated a priori but determined upon feasible size. Data analyses were conducted using JMP statistical software version 13.1.0 (SAS Institute Inc., Cary, NC, USA). All statistical tests were two-tailed, and the chosen type 1 error rate was *p* <  0.05.

## Results

**Fig. 1:**
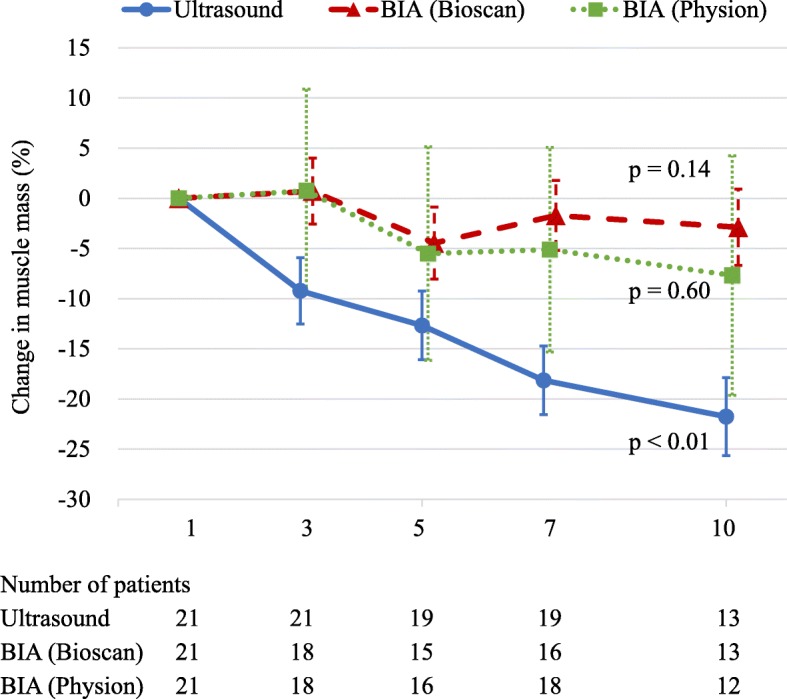
Percentage change in muscle mass, monitored by an ultrasound and two BIAs. Percentage change in muscle mass was calculated in the comparison with the value of day 1. Muscle mass, monitored by an ultrasound, statistically significantly (*p* <  0.01) decreased, whereas muscle mass, monitored by two BIAs, did not statistically significantly decrease (Bioscan, *p* = 0.14; Physion, *p* = 0.60). *p* values were derived from mixed-effects models for repeated measures. Data are presented as means and 95% confidence intervals. The number of patients daily is displayed below the graph. BIA bioelectrical impedance analysis

Twenty-one patients were enrolled, and all patients participated in the study on day 3, 19 patients on day 5, 19 on day 7, and 13 on day 10 (Fig. 1). The age of the patients was 69 (interquartile range, IQR, 59–74) years, and 16 patients were men (patient characteristics are summarized in Table 1). The APACHE II score was 28 (IQR, 23–30). The duration of mechanical ventilation was 7 days (IQR, 5–18 days). The reasons for admission were surgical (29%) and non-surgical (71%).

**Table 1:** Patients’ characteristics (*n* = 21)

Variables	Overall
Age, years	69 (59–74)
Gender (men), *n* (%)	16 (76%)
Body mass index, kg/m^2^ (mean (SD))	22.9 ± 3.8
APACHE II score	28 (23–30)
ICU length of stay, days	11 (9–25)
Duration of mechanical ventilation, days	7 (5–18)
Hospital length of stay, days	43 (26–117)
Mortality in the ICU, *n* (%)	6 (29%)
ICU admission reasons	
Surgical, *n* (%)	6 (29%)
Non-surgical, *n* (%)	15 (71%)

Data were presented as median (IQR) unless otherwise indicated. *SD* standard deviation, *APACHE* Acute Physiology and Chronic Health Evaluation, *ICU* intensive care unit, *IQR* interquartile range

### Percentage changes in muscle mass

We analyzed 93 ultrasound images in 21 patients. The muscle mass decreased progressively by 9.2% (95% CI, 5.9–12.5%), 12.7% (95% CI, 9.3–16.1%), 18.2% (95% CI, 14.7–21.6%), and 21.8% (95% CI, 17.9–25.7%) on days 3, 5, 7, and 10, respectively (*p* <  0.01; MMRM, Fig. 1). Using Bioscan, 83 tests in 21 patients were analyzed. Ten tests were missing data (7 tests were measurement errors and 3 tests were unable to be performed). The muscle mass had changed by 0.7% (95% CI, − 2.6 to 4.0%), − 4.5% (95% CI, − 8.1 to − 0.9%), − 1.7% (95% CI, − 5.2 to 1.8%), and − 2.9% (95% CI, − 6.7 to 0.9%) on days 3, 5, 7, and 10, respectively (*p* = 0.14; MMRM). Using Physion, 8 tests were missing data (5 tests were measurement errors and 3 tests were unable to be performed). In 85 measurements, the muscle mass had changed by 0.7% (95% CI, − 9.4% to 10.9%), − 5.5% (95% CI, − 16.2% to 5.1%), − 5.1% (95% CI, − 15.3% to 5.1%), and − 7.7% (95% CI, − 19.6 to 4.3%) on days 3, 5, 7, and 10, respectively (*p* = 0.60; MMRM).

### Relationship between ultrasound and BIA in muscle mass evaluation

**Fig. 2:**
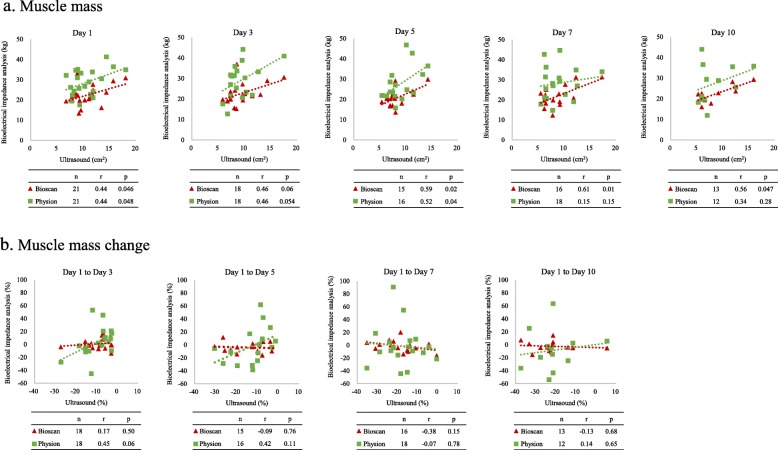
Relationship between ultrasound and BIA in muscle mass evaluation. The correlation between ultrasound and two BIAs. **a** Muscle mass. **b** Muscle mass change. **a** Muscle mass was compared at one point in each measurement day. BIA was correlated with ultrasound in some measurement days (Bioscan, days 1, 5, 7, and 10; Physion, days 1 and 5). **b** Muscle mass change was compared from day 1 to each measurement day. The percentage change of muscle mass in BIA did not correlate with the findings using ultrasound. The correlations were shown under every graph. Pearson correlation coefficient was used to investigate the relationships. BIA bioelectrical impedance analysis

In muscle mass assessment at one point, BIA was correlated with ultrasound in some examination days (Bioscan, days 1, 5, 7, and 10; Physion, days 1 and 5, Fig. 2a). In sum, average correlation was *r* = 0.51, *p* <  0.01 in Bioscan (*n* = 83) and *r* = 0.37, *p* <  0.01 in Physion (*n* = 85). In contrast, the percentage change of muscle mass in BIA did not correlate with the findings using ultrasound (Fig. 2b). The average correlation was *r* = − 0.05, *p* = 0.69 in Bioscan (*n* = 62) and *r* = 0.23, *p* = 0.07 in Physion (*n* = 64).

### Relationship among ultrasound, BIA, and CT in muscle mass evaluation

**Fig. 3:**
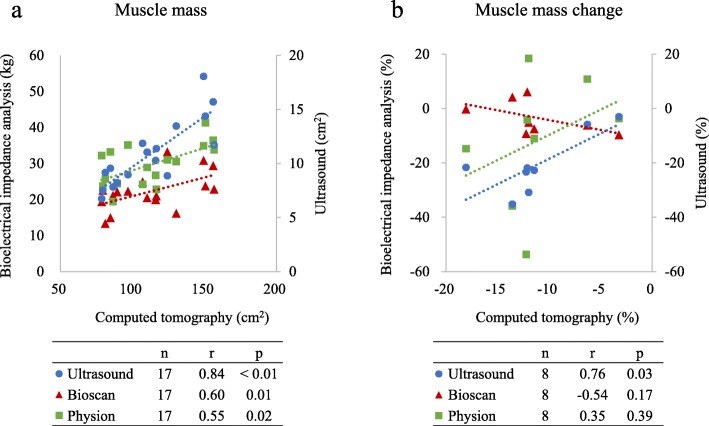
Relationship among ultrasound, BIA, and CT in muscle mass evaluation. The correlation among ultrasound, BIA, and CT. **a** Muscle mass. **b** Muscle mass change. **a** Muscle mass was compared in 17 patients at day 1. Both ultrasound and BIA were correlated with CT measurements. **b** Muscle mass change was compared from day 1 to each measurement day (1, 2, 3, and 2 patients at days 3, 5, 7, and 10). Ultrasound, not BIA, was correlated with CT measurements. The correlations were shown under both graphs. Pearson correlation coefficient was used to investigate the relationships. BIA bioelectrical impedance analysis, CT computed tomography

We analyzed 17 CT images in 17 patients at day 1 and 8 patients with the subsequent examination (1, 2, 3, 2 patients at days 3, 5, 7, 10). At day 1, ultrasound showed a strong correlation with CT measurement (*r* = 0.84, *p* <  0.01, Fig. 3a). BIA was moderately correlated with CT measurement (Bioscan, *r* = 0.60, *p* = 0.01; Physion, *r* = 0.55, *p* = 0.02). The percentage change of muscle mass in CT correlated only with ultrasound (ultrasound, *r* = 0.76, *p* = 0.03; Bioscan, *r* = − 0.54, *p* = 0.17; Physion, *r* = 0.35, *p* = 0.39, Fig. 3b).

### Relationship against fluid balance

In ultrasound, the percentage change of muscle mass was not correlated with interval fluid balance in the ICU (*r* = 0.04, *p* = 0.74), whereas subcutaneous tissue was correlated with interval fluid balance (*r* = 0.38, *p* <  0.01, Table 2, Additional file 1: Fig. S5). The measurements of BIA and body weight change were positively correlated with interval fluid balance (*r* = 0.34–0.57, *p* <  0.01). In accumulated fluid balance, fluid shift and body weight were also correlated (*r* = 0.29–0.57, *p* ≦ 0.02, Additional file 1: Fig. S6). In ultrasound, the change of muscle mass was not correlated with accumulated fluid balance in the ICU (*r* = 0.16, *p* = 0.18), and subcutaneous tissue was also not correlated with accumulated fluid balance (*r* = 0.20, *p* = 0.09). Furthermore, the muscle mass assessment of Bioscan was not correlated with accumulated fluid balance, (*r* = 0.15, *p* = 0.25), although it was correlated at day 3 (*r* = 0.69, *p* <  0.01).

**Table 2:** Relationship against fluid balance (interval and accumulated)

Percentage change (%)	Interval			Accumulated		
	*n*	*r*	*p* value	*n*	*r*	*p* value
Muscle mass						
Ultrasound	72	0.04	0.74	72	0.16	0.18
BIA (Bioscan)	55	0.37	< 0.01	62	0.15	0.25*
BIA (Physion)	60	0.51	< 0.01	64	0.45	< 0.01
Subcutaneous tissue by ultrasound	72	0.38	< 0.01	72	0.20	0.09
Fluid shift measured by BIA (Bioscan)	55	0.56	< 0.01	62	0.56	< 0.01
Extracellular water	55	0.55	< 0.01	62	0.57	< 0.01
Intracellular water	55	0.47	< 0.01	62	0.29	0.02
Fluid shift measured by BIA (Physion)	60	0.34	< 0.01	64	0.34	< 0.01
Body weight	67	0.57	< 0.01	65	0.43	< 0.01

Interval: all variables are the changes (%) between each measurement day. Accumulated: all variables are the changes (%) from day 1 to measurement days. The Pearson correlation coefficient was used to investigate the relationships. *BIA* bioelectrical impedance analysis

*The correlation of Bioscan was significant only at day 3 (*r* = 0.69, *p* < 0.01 at day 3, *r* = − 0.06, *p* = 0.83 at day 5, *r* = 0.18, *p* = 0.51 at day 7, *r* = − 0.12, *p* = 0.71 at day 10)

## Discussion

In this observational study, we found muscle mass monitoring by BIA was complicated by the fluid shift and could not monitor the change of muscle mass in critically ill patients, although muscle mass assessment at one point moderately correlated with ultrasound and CT. In contrast, the use of ultrasound monitored progressive muscle atrophy over the ICU stay without the influence of fluid shift. To our knowledge, this is the first study to investigate muscle mass monitoring capacity by BIA and ultrasound with the assessment of fluid balance.

In our study, the reference standard of muscle mass evaluation was based on ultrasound. The accuracy of muscle mass evaluation by ultrasound has been established in many researches [1, 8]. In this study, days over ICU stay resulted in an atrophy rate similar to the one previously reported (21.8% on day 10 in our study vs. 17.7% in Puthucheary’s study, and vs. 29.9% in Parry’s study) [1, 8]. Furthermore, we found that muscle mass assessment by ultrasound was strongly correlated with CT (*r* = 0.76–0.84, *p* ≤ 0.03). Because CT can separate muscle, fat, and other tissues, CT is considered as an accurate and precise method for muscle mass assessment [9]. Therefore, the value of ultrasound assessment is reliable.

In a previous study, Kim et al. found that muscle mass assessment by using BIA correlated with CT assessment (*n* = 135, *r* = 0.58–0.73, *p* <  0.0001), suggesting that BIA can be used for muscle mass assessment of critically ill patients [5]. Another previous study by Kuchnia et al. reported that phase angle and impedance ratio measured by BIA correlated with muscle mass in CT (*n* = 71, *r* = 0.67–0.78, *p* <  0.05) [7]. Consistent with these findings, muscle mass assessment by BIA was moderately correlated at one point in our study. However, no study investigated the usefulness of BIA for the muscle mass monitoring in the same critically ill patients. Hosono et al. successfully used BIA for follow-up measurements of muscle mass with rheumatologic patients [10], but its use in critically ill patients is still unknown because patients in the ICU are exposed to abnormal fluid status. In the edematous condition, the use of BIA for muscle mass monitoring may overestimate the muscle mass in critically ill patients. In a previous study by Kim et al., BIA measurement was complicated by edema and overestimated muscle mass in critically ill patients. Extracellular-to-total body water (ECW/TBW) is often used for the assessment of edema with the cutoff value of 0.40 [11]. In this study, the median extracellular-to-total body water (ECW/TBW) was 0.48 (IQR, 0.46–0.51). Similarly, in previous reports, most critically ill patients had increased amount of extracellular fluid [5, 11]. Therefore, the results of BIA for muscle mass monitoring should be carefully interpreted in critically ill patients.

However, the estimation of muscle mass may differ among BIA devices [12]. In this two-device comparison study, Physion was possibly more influenced by fluid balance because it had greater CI (Fig. 1) and higher correlation with fluid balance than Bioscan (Physion, *r* = 0.45–0.51; Bioscan, *r* = 0.15–0.37). In Bioscan, the muscle mass assessment was not influenced by accumulated fluid balance at the later stage in the ICU. We cannot conclude our results can be applied to all the BIA devices because Inbody S10 (InBody Corp, South Korea) or MF-BIA QuadScan 4000 (Bodystas LTD, United Kingdom) were used in previous studies [5, 7].

Edema is also of concern for the muscle mass evaluation by ultrasound. Intramuscular edema may complicate muscle mass evaluation. In our study, the interval fluid balance affected subcutaneous thickness and did not affect the muscle mass significantly, implying that the increased amount of water may increase extracellular water and accumulate in the subcutaneous tissue. Cartwright’s study, the thickness of subcutaneous tissue increased over the ICU stay, and the results indicated that the ultrasound of the subcutaneous tissue may detect edema in ICU patients [13]. Similarly, Campbell found muscle thickness correlated with fat-free mass in edematous patients, and speculated that most fluid was not retained in the body of muscle [14]. In our research, subcutaneous thickness was associated with interval fluid balance, not accumulated fluid balance. In prolonged ICU stay, subcutaneous tissue thickness is influenced by the change of adipose tissue and muscle mass. Therefore, interval fluid balance was more sensible to the change of edema. Although the influence of edema needs further investigation, our results indicate that ultrasound is suitable for the muscle mass monitoring in edematous critically ill patients.

There are advantages and limitations among measurement methods. CT, MRI, and dual-energy X-ray absorptiometry may be more accurate in evaluating muscle mass, but critically ill patients need to be transferred to the examination room with some risks to perform these methods. In contrast, ultrasound and BIA can be used noninvasively at the bedside. Because BIA does not need measurement skills and can easily be used by any operator, it is clinically useful if we can apply the device for muscle mass monitoring. However, the result of this study was contrary to the use for monitoring. We may need to clarify some formula to use BIA in critically ill patients because muscle mass estimation was derived from healthy volunteers in most BIAs [15]. Conclusively, in our research, ultrasound was more useful for muscle mass monitoring in the ICU although it will need measurement skill for accurate measurements. Acquisition of ultrasound measurement skill may be necessary for muscle mass monitoring for better nutritional and metabolic support during critical illness.

Our findings suggest that it is worthwhile to use ultrasound for muscle mass monitoring. Although BIA may be useful to assess muscle mass at one point, the results of BIA for muscle mass monitoring should be carefully interpreted in critically ill patients. To improve nutritional support and rehabilitation, further evidence is needed for the monitoring of muscle mass in critically ill patients.

## Limitations

Our study has several limitations. First, the study has a small sample size in a single center. Particularly, the number of CT scan was limited due to the retrospective nature to avoid extra radiation exposure; consequently, our observations need to be validated by study of a larger population. Second, some data on BIA measurement were missing mostly due to the measurement error, whereas ultrasound measurement did not have missing data.

## Conclusions

We evaluated muscle mass monitoring methods in critically ill patients and found that ultrasound is suitable for sequential monitoring of muscle atrophy. Monitoring by BIA should be carefully interpreted due to the fluid change.

## Supplementary information

**Additional file 1: Table S1.** Reproducibility of measurements. **Figure S1.** Ultrasound sites for the upper and lower limbs. (a) Biceps brachii muscle was measured at two-thirds of the way between the acromion and the antecubital crease. (b) Rectus femoris muscle was measured at midway between the anterior superior iliac spine and the proximal end of the patella. **Figure S2.** Ultrasound image of muscle and subcutaneous tissue. (a) The cross-sectional area of biceps brachii was measured by outlining the muscle area shown in the transverse plane. (b) The cross-sectional area of rectus femoris was measured by outlining the muscle area shown in the transverse plane. (c) Subcutaneous tissue thickness of biceps brachii was defined as depth between the skin and the superficial fascia of the biceps brachii muscle. (d) Subcutaneous tissue thickness of rectus femoris was defined as depth between the skin and the superficial fascia of the rectus femoris muscle. **Figure S3.** CT image of muscle. Muscle mass area was evaluated from computed tomography at the L3 spine level by using image J software (National Institutes of health, Bethesda, MD, USA). **Figure S4.** Fluid balance calculation. The upper side depicts interval fluid balance between measurement days. On the other hand, the lower side depicts accumulated fluid balance from day 1 to measurement days. **Figure S5.** Relationship between measurements and interval fluid balance. Interval fluid balance was compared with variable measurements between each measurement day. **Figure S6.** Relationship between measurements and accumulated fluid balance. Accumulated fluid balance was compared with variable measurements from day 1 to measurement days.

## Data Availability

The datasets used and/or analyzed during the current study are available from the corresponding author on reasonable request.

## Abbreviations

APACHE II: Acute physiology and chronic health evaluation II
BIA: Bioelectrical impedance analysis
CI: Confidence interval
CT: Computed tomography
ECW: Extracellular water
ICU: Intensive care unit
IQR: Interquartile range
MMRM: Mixed-effects models for repeated measures
MRI: Magnetic resonance imaging
TBW: Total body water
