# Supplementary material for: Monitoring of muscle mass in critically ill patients: comparison of ultrasound and two bioelectrical impedance analysis devices
Source: J Intensive Care. 2019 Dec 16;7:61. doi: 10.1186/s40560-019-0416-y (PMC6916000; doi:10.1186/s40560-019-0416-y)
Supplement: Supplementary file 1 — Additional file 1: Table S1. Reproducibility of measurements. Figure S1. Ultrasound sites for the upper and lower limbs. (a) Biceps brachii muscle was measured at two-thirds of the way between the acromion and the antecubital crease. (b) Rectus femoris muscle was measured at midway between the anterior superior iliac spine and the proximal end of the patella. Figure S2. Ultrasound image of muscle and subcutaneous tissue. (a) The cross-sectional area of biceps brachii was measured by outlining the muscle area shown in the transverse plane. (b) The cross-sectional area of rectus femoris was measured by outlining the muscle area shown in the transverse plane. (c) Subcutaneous tissue thickness of biceps brachii was defined as depth between the skin and the superficial fascia of the biceps brachii muscle. (d) Subcutaneous tissue thickness of rectus femoris was defined as depth between the skin and the superficial fascia of the rectus femoris muscle. Figure S3. CT image of muscle. Muscle mass area was evaluated from computed tomography at the L3 spine level by using image J software (National Institutes of health, Bethesda, MD, USA). Figure S4. Fluid balance calculation. The upper side depicts interval fluid balance between measurement days. On the other hand, the lower side depicts accumulated fluid balance from day 1 to measurement days. Figure S5. Relationship between measurements and interval fluid balance. Interval fluid balance was compared with variable measurements between each measurement day. Figure S6. Relationship between measurements and accumulated fluid balance. Accumulated fluid balance was compared with variable measurements from day 1 to measurement days. [file 40560_2019_416_MOESM1_ESM.docx]

Supplemental File

Monitoring of muscle mass in critically ill patients

|  | Correlation coefficient | | Bland-Altman 95% LOA | |
| --- | --- | --- | --- | --- |
| Variables | R^2^ | p | Bias | 95% LOA |
| Intraobserver reproducibility |  |  |  |  |
| Cross-sectional area |  |  |  |  |
| Biceps brachii muscle | 0.93 | < 0.01 | 0.028 ± 0.094 | -0.184 to 0.240 |
| Rectus femoris muscle | 0.99 | < 0.01 | 0.113 ± 0.067 | -0.039 to 0.265 |
| Subcutaneous tissue |  |  |  |  |
| Biceps brachii muscle | 0.83 | < 0.01 | 0.063 ± 0.065 | -0.084 to 0.210 |
| Rectus femoris muscle | 0.99 | < 0.01 | 0.128 ± 0.092 | -0.080 to 0.335 |
| Computed tomography | 0.90 | < 0.01 | 0.130 ± 2.165 | -4.768 to 5.028 |
| Interobserver reproducibility |  |  |  |  |
| Cross-sectional area |  |  |  |  |
| Biceps brachii muscle | 0.93 | < 0.01 | 0.113 ± 0.069 | 0.094 to 0.240 |
| Rectus femoris muscle | 0.98 | < 0.01 | 0.113 ± 0.067 | -0.039 to 0.265 |
| Subcutaneous tissue |  |  |  |  |
| Biceps brachii muscle | 0.90 | < 0.01 | 0.075 ± 0.054 | -0.048 to 0.198 |
| Rectus femoris muscle | 0.99 | < 0.01 | 0.187 ± 0.115 | -0.072 to 0.446 |
| Computed tomography | 0.91 | < 0.01 | -1.050 ± 2.030 | -5.642 to 3.541 |

**Table S1** Reproducibility of measurements

**Reproducibility was assessed for 10 critically ill patients. The Pearson correlation coefficient and Bland-Altman plot were determined by using JMP statistical software version 13.1.0 (SAS Institute Inc., Cary, NC, USA).**

**LOA: limits of agreement**


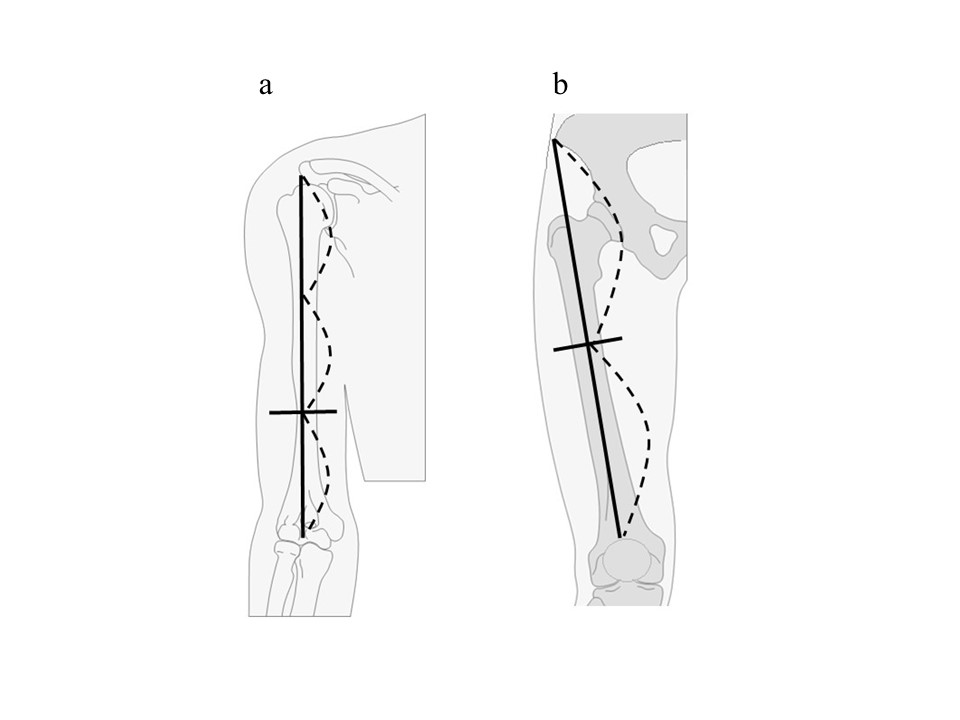


**Figure S1** Ultrasound sites for the upper and lower limbs

(a) Biceps brachii muscle was measured at two-thirds of the way between the acromion and the antecubital crease. (b) Rectus femoris muscle mass was measured at midway between the anterior superior iliac spine and the proximal end of the patella.


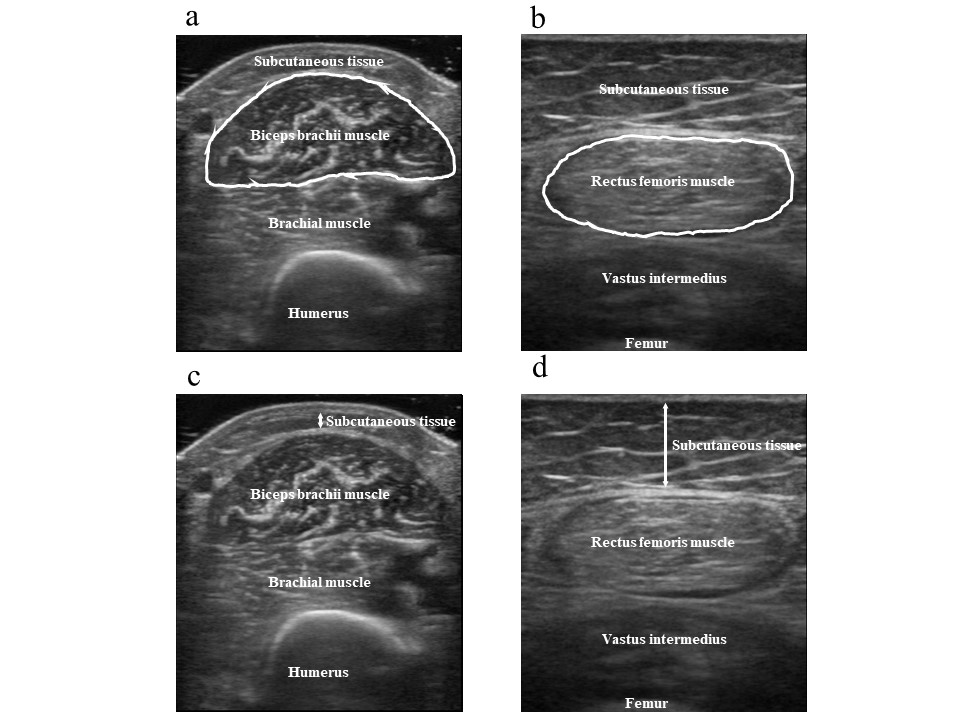
**Figure S2** Ultrasound image of muscle and subcutaneous tissue

(a) The cross-sectional area of biceps brachii was measured by outlining the muscle area shown in the transverse plane. (b) The cross-sectional area of rectus femoris was measured by outlining the muscle area shown in the transverse plane. (c) Subcutaneous tissue thickness of biceps brachii was defined as depth between the skin and the superficial fascia of the biceps brachii muscle. (d) Subcutaneous tissue thickness of rectus femoris was defined as depth between the skin and the superficial fascia of the rectus femoris.


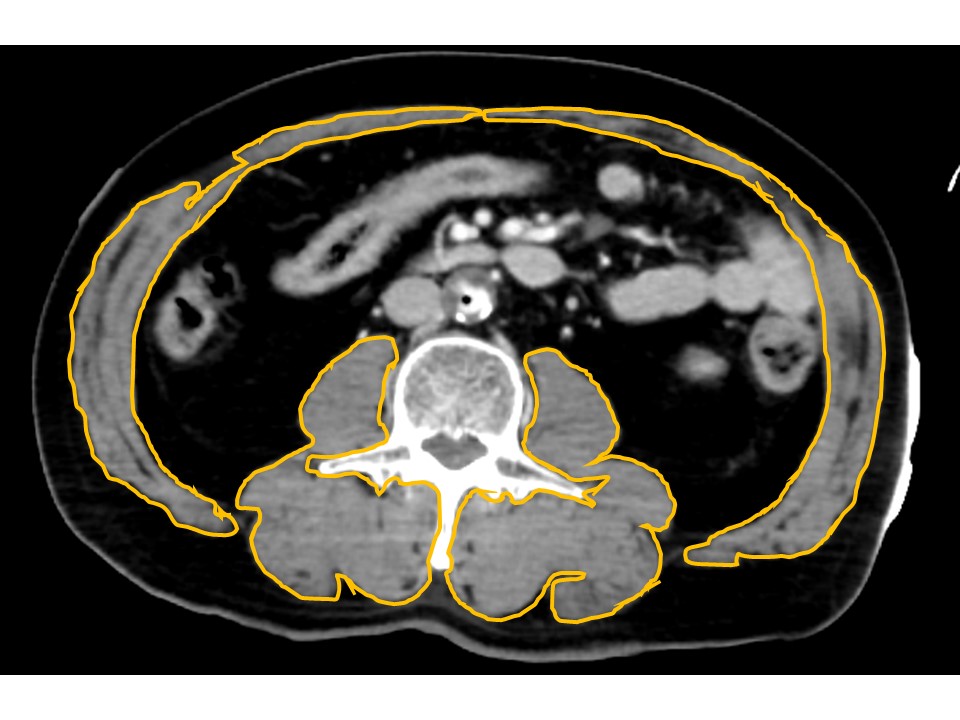
**Figure S3** CT image of muscle

Muscle mass area was evaluated from computed tomography at the L3 spine level by using image J software (National Institutes of health, Bethesda, MD, USA).

**
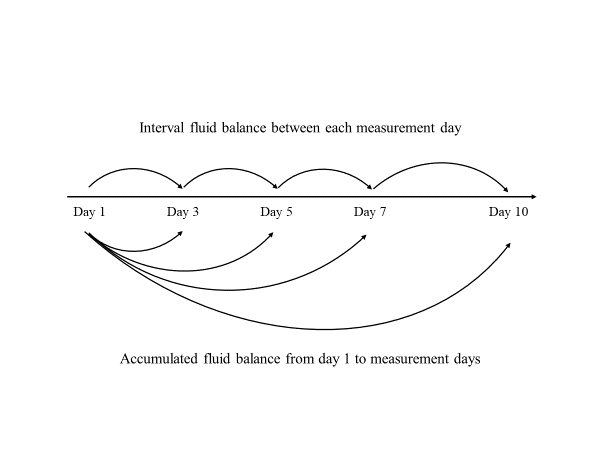
**

**Figure S4** Fluid balance calculation

The upper side depicts interval fluid balance between measurement days. On the other hand, the lower side depicts accumulated fluid balance from day 1 to measurement days.


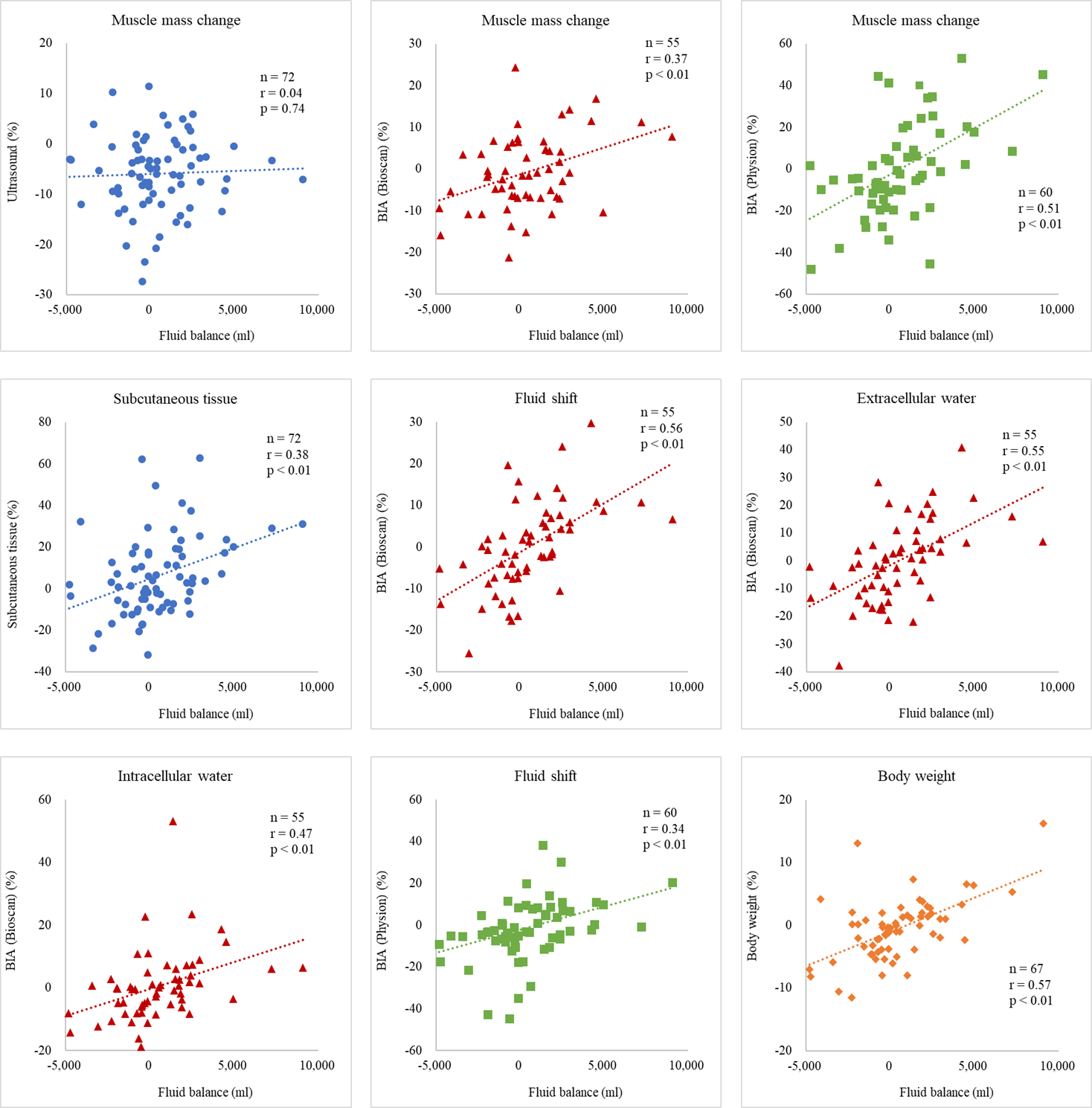


**Figure S5** Relationship between measurements and interval fluid balance.

Interval fluid balance was compared with variable measurements between each measurement day.


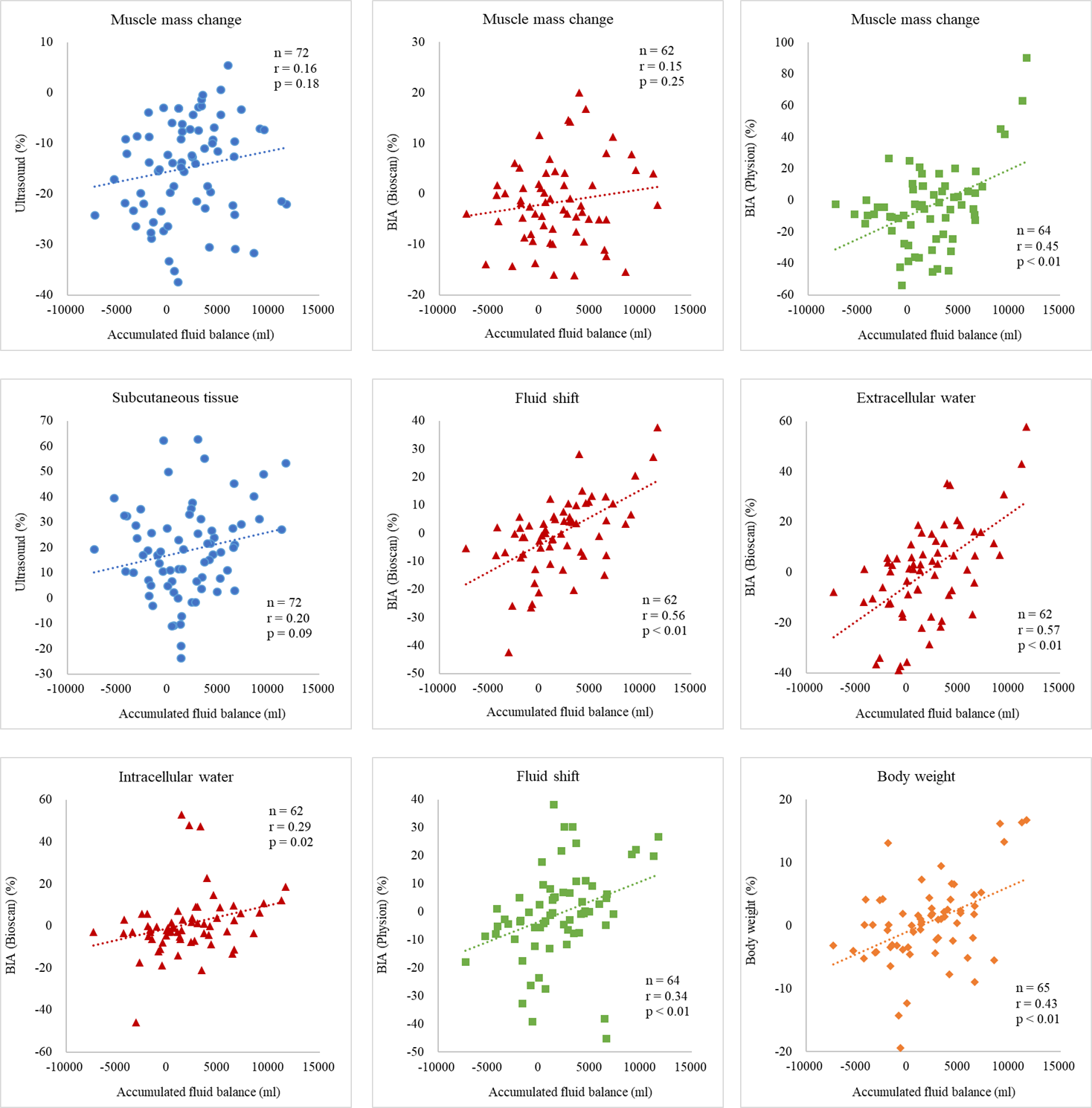


**Figure S6** Relationship between measurements and accumulated fluid balance

Accumulated fluid balance was compared with variable measurements from day 1 to measurement days.
